# *C. elegans patched-3* is an essential gene implicated in osmoregulation and requiring an intact permease transporter domain

**DOI:** 10.1016/j.ydbio.2010.12.035

**Published:** 2011-03-15

**Authors:** Alexander Soloviev, Joseph Gallagher, Aline Marnef, Patricia E. Kuwabara

**Affiliations:** University of Bristol, School of Biochemistry, Bristol UK BS8 1TD

**Keywords:** Patched, Hedgehog, Transporter, Permease, Sterol sensing domain

## Abstract

The nematode *Caenorhabditis elegans* has retained a rudimentary Hedgehog (Hh) signalling pathway; Hh and Smoothened (Smo) homologs are absent, but two highly related *Patched* gene homologs, *ptc-1* and *ptc-3*, and 24 *ptc-related* (*ptr)* genes are present. We previously showed that *ptc-1* is essential for germ line cytokinesis. Here, we report that *ptc-3* is also an essential gene; the absence of *ptc-3* results in a late embryonic lethality due to an apparent defect in osmoregulation. Rescue of a *ptc-3* mutant with a *ptc-3::gfp* translational reporter reveals that *ptc-3* is dynamically expressed in multiple tissues across development. Consistent with this pattern of expression, *ptc-3*(*RNAi*) reveals an additional postembryonic requirement for *ptc-3* activity. Tissue-specific promoter studies indicate that hypodermal expression of *ptc-3* is required for normal development. Missense changes in key residues of the sterol sensing domain (SSD) and the permease transporter domain GxxxD/E motif reveal that the transporter domain is essential for PTC-3 activity, whereas an intact SSD is dispensable. Taken together, our studies indicate that PTC proteins have retained essential roles in *C. elegans* that are independent of Smoothened (Smo). These observations reveal novel, and perhaps ancestral, roles for PTC proteins.

## Research Highlights

► *C. elegans* lacks conventional Hedgehog signalling, yet *ptc-3* is an essential gene. ► Deletion of *ptc-3* causes a lethal defect in osmoregulation. ► *ptc-3* epidermal expression is necessary and sufficient for viability. ► An intact sterol sensing domain is dispensable for *ptc-3* activity. ► Missense mutations highlight the importance of the *ptc-3* transporter domain.

## Introduction

The importance of the Hedgehog (Hh) signal transduction pathway has been well established in both invertebrates and vertebrates (McMahon et al., 2003; Varjosalo et al., 2006; Jiang and Hui, 2008). Hh is a secreted morphogen that controls cell patterning of the embryo, limb bud and neural tube, organ formation, and cell proliferation. Human pathologies and cancers have been shown to arise when genes in this pathway are dysregulated through familial or sporadic mutation (Barakat et al., 2010).

Hh proteins are bipartite; the C-terminal domain promotes autoproteolytic cleavage to yield an N-terminal peptide (Hh-N) with signalling activity (Mann and Beachy, 2004). Hh-N is further modified by the addition of cholesterol to the C-terminus and palmitic acid to the N-terminus. The secretion of Hh from a signalling cell is facilitated by Dispatched (Disp) (Burke et al., 1999; Ma et al., 2002; Nakano et al., 2004), a protein that shares extensive sequence and topological similarities with Patched (Ptc) proteins (Hooper and Scott, 1989; Nakano et al., 1989), which are 12-pass membrane-spanning receptors for Hh (Marigo et al., 1996; Stone et al., 1996). Ptc inhibits the activity of the Smoothened (Smo) G-protein-coupled membrane receptor, which is related to the Frizzled family of Wnt receptors (Alcedo et al., 1996; Stone et al., 1996; van den Heuvel and Ingham, 1996), by preventing Smo from localizing to the plasma membrane in *Drosophila* or to the primary cilium in vertebrates where it is active (Huangfu et al., 2003; Rohatgi et al., 2007). However, binding of Hh to the Ptc receptor allows Smo to activate a signal transduction cascade, which culminates in the transcriptional induction of target genes, such as growth factors, by the zinc finger transcription factor Ci in *Drosophila*, or by the orthologous Gli proteins in vertebrates (Alexandre et al., 1996; Bai et al., 2004). Ptc also acts as a sink, which limits the range of Hh signalling (Chen and Struhl, 1996). The Ci and Gli proteins are subject to multiple levels of regulation; in the absence of Hh-mediated signalling, repressive forms of these factors are produced by proteolysis (Kalderon, 2005).

In *Caenorhabditis elegans*, whole genome sequence analysis has revealed the existence of a rudimentary Hh signalling pathway (Bürglin and Kuwabara, 2006). Surprisingly, genes encoding Hh and Smo are absent, although the worm encodes over 60 predicted Hedgehog-like proteins, which are collectively referred to as the Hh-related (Hh-r) proteins (Aspock et al., 1999; Bürglin and Kuwabara, 2006). Many of the *C. elegans* Hh-r proteins share in common the hallmark bipartite structure of Hh, but none has extensive sequence similarity with the Hh-N signalling moiety. At least one of the Hh-related proteins, WRT-1, is capable of undergoing autoproteolytic cleavage in *Drosophila* S2 cells (Porter et al., 1996); however, the signalling potential of Hh-r proteins has not yet been fully investigated.

Despite the absence of Hh and Smo, the worm has 3 *ptc* and 24 *ptr* (for *pt*c **r**elated) genes, which encode proteins that share extensive sequence similarity with PTC proteins from *Drosophila* and vertebrates (Kuwabara et al., 2000). In common with other Ptc family members, the *C. elegans* PTC and PTR proteins carry a sterol-sensing domain (SSD) and a permease domain associated with RND (Resistance–nodulation–division) efflux transporters. These domains are also present in Disp and the disease-related Niemann-Pick Type C (NPC1) protein (Kuwabara and Labouesse, 2002; Eaton, 2008). In *C. elegans*, two Disp gene orthologs, *che-14/ptd-1* and *ptd-2*, have also been identified (Kuwabara et al., 2000; Michaux et al., 2000). Phenotypic and ultrastructural analyses of *che-14* mutants show that the CHE-14 protein promotes polarized apical secretion from epidermal and support cells (Michaux et al., 2000). An ortholog of the Ci and Gli proteins, named TRA-1, is also present in *C. elegans*. In worms, TRA-1 is the master regulator of the signal transduction pathway controlling sexual cell fate decisions in somatic tissues (Zarkower, 2006). Interestingly, the mechanisms controlling Ci/Gli regulation have been partially conserved in *C. elegans*. In particular, it has been shown that TRA-1A is proteolytically processed to generate truncated phosphoisoforms that repress the transcription of genes promoting male development (Schvarzstein and Spence, 2006; Starostina et al., 2007). In addition, TRA-2A, a transmembrane receptor that promotes female development, shares marginal sequence and topological similarities with Ptc (Kuwabara et al., 2000).

In *C. elegans*, it has been possible to address whether Ptc proteins have roles that are independent of their interactions with Hh and Smo. Several studies indicate that despite the absence of Hh and Smo, many of the *ptc, ptr* and a subset of *hh-*r genes are needed during *C. elegans* development (Kuwabara et al., 2000; Zugasti et al., 2005; Hao et al., 2006a; Hao et al., 2006c). Notably, we have shown that *ptc-1* plays an essential role in germ line cytokinesis (Kuwabara et al., 2000). A *ptc-2* gene also exists, which shares > 95% identity with *ptc-1* at the nucleotide level. However, *ptc-2* appears to be a *C. elegans* pseudogene that arose after a recent partial duplication of the *ptc-1* locus, an event that must have occurred after *C. elegans* diverged from the closely related nematodes *C. briggsae* and *C. remanei* (Kuwabara et al., 2000).

To gain further insights into the novel or ancestral functions of the Patched proteins, we have cloned and analyzed the role of the *ptc-3* gene in *C. elegans* development. We show that *ptc-3* is an essential gene, which is required during late embryogenesis and again during postembryonic stages of *C. elegans* development. To understand the postembryonic role of *ptc-3*, we have analyzed its temporal and spatial pattern of expression. Our analysis reveals that *ptc-3* expression is highly dynamic; PTC-3 is present in multiple tissues throughout development. We further demonstrate that hypodermal PTC-3 expression is sufficient to rescue the lethality of a *ptc-3* deletion mutant. We have also investigated the relative importance of the SSD and permease transporter domain for maintaining PTC-3 function.

## Materials and methods

### Worm strains and culture

Worms were grown on petri dishes or in liquid as described (Sulston and Hodgkin, 1988). All genetic characterizations were performed at 20 °C using worms that were not starved or recovering from the dauer state. To obtain a synchronized population of L1 larvae, eggs were isolated after hypochlorite treatment of gravid hermaphrodites and cultured in M9 buffer in the absence of food (Wood and Researchers, 1988).

### Phenotypic characterization of ptc-3

RNAi was initially performed by microinjecting *ptc-3* dsRNA prepared using the Megascript kit (Ambion) into the germ line syncytium (Stephens et al., 2004). To analyze postembryonic phenotypes associated with the knockdown of *ptc-3* activity, *ptc-3*(*RNAi*) was performed by feeding worms the bacterial strain HT115 carrying the pPK463 plasmid, which contains ~ 400 bp of *ptc-3* cDNA sequence (Timmons et al., 2001; Stephens et al., 2004). To overcome the late embryonic lethality associated with the absence of *ptc-3*, RNAi was also initiated using L1 staged N2 animals, which were obtained by bleaching gravid adult hermaphrodites and allowing embryos to develop in the absence of food. Weaker RNAi effects were obtained by mixing RNAi feeding bacteria with OP50.

A *ptc-3*(*ok121*) deletion mutant was obtained from the *C. elegans* Gene Knockout Consortium and outcrossed 5x prior to study. The *ptc-3*(*ok121*) deletion was minimally balanced *in trans* with *sup-9*(*n180*) by generating the strain *ptc-3*(*ok121*)*/sup-9*(*n180*)*; unc-93*(*e1500sd*). *sup-9*(*n180*) is a recessive suppressor that restores wild-type movement to *unc-93*(*e1500sd*) rubber-band mutants (Greenwald and Horvitz, 1980). Broods derived from *ptc-3*(*ok121*)/+ heterozygous mothers (P_0_) were scored by transferring the P_0_ mother daily to a fresh plate until egg production ceased; dead eggs, which failed to hatch, were counted 24 h after transfer of the mother. The *ptc-3* deletion allele was detected by performing single-worm PCR using the primers ptc-3_24c and ptc-3_6c, which span the deletion breakpoints (see below) to generate a 1.051 kb PCR deletion product and a 3.045 kb wild-type fragment.

### Identification of ptc-3(ok121) deletion breakpoints

A 1.05 kb PCR product spanning the *ptc-3*(*ok121*) deletion was amplified using the oligonucleotides ptc-3_23c and ptc-3_5c (see below). The resulting product was sequenced using the primers ptc-3_24c and ptc-3_6c; the deletion breakpoint was found to be flanked by the sequences: GGATTCGCTACTCATCTTGGGATCAATTTC CAGGATTAGGCAAAGGCTTAGGCTTCAGCT.

### Molecular characterization of ptc-3

A YAC, Y53C11, carrying the *ptc-3* locus was identified by hybridizing a YAC grid (kindly provided by Ratna Shownkeen) with a ^32^P-labelled random primed PCR fragment amplified from the *ptc-3* cDNA sequence yk68c12 (kindly provided by Yuji Kohara) using the primers ptc-3_311F and ptc-3_262R. The same ^32^P-labelled *ptc-3* cDNA probe was also hybridized to a fosmid grid; nine fosmids were identified: H05G20, H16N07, H22H09, H23D21; H26D12, H35J09, H35P10, H36N03, and H39E13. Plasmid libraries carrying *ptc-3* genomic sequence were generated by digesting fosmids H35N03 and H35P10 with EcoRI or EagI and sub-cloning their inserts in the vector pBSIIKS(+) (Stratagene).

cDNA clones were obtained as a kind gift from Yuji Kohara and were also generated by performing RT-PCR. The presence of a trans splice leader sequence was shown by priming first strand cDNA from N2 poly(A) + mRNA using the ptc-3_14c oligonucleotide with reverse transcriptase (Life Sciences); double-stranded cDNA was generated by PCR with primers ptc-3_15c and SL1. cDNAs sequences were obtained by from the sequencing service at the University of Dundee or Eurofins.

### Generation of transgenic *C. elegans*

Transgenic nematodes were generated by the standard technique of co-injecting 5–10 ng/μl of test plasmid with 80 ng/μl pRF4 [*rol-6*(*su1006*)], which confers a dominant Rol phenotype (Mello and Fire, 1995); alternatively, 50 ng/μl of pTG96, which expresses the nuclear marker (NLS) *sur-5::gfp* (Gu et al., 1998), or 20 ng/μl punc-122::mRFP, which expresses a mRFP protein scavenged by coelomocytes, were co-injected to provide markers indicative of co-transformation. The primer pair ptc-3_12c and ptc-3_24c was used to determine the *ptc-3* genotype of transgenic animals by single-worm PCR. The DNA sequence amplified by these primers is absent in the *ok121* deletion mutant; however, this primer pair produces a 1.086 kb PCR product corresponding to the wild-type *ptc-3* locus and a larger 2.082 kb product corresponding to the *gfp* cassette inserted in pPK348. The *ptc-3* genotype and the presence of other *ptc-3* transgenes were determined using a similar strategy but different primer pairs.

### Transgenic rescue of ptc-3(ok121)

pPK347 carries a 12.29 kb *C. elegans* genomic DNA insert including the putative promoter, coding region and 3′ UTR of *ptc-3*. This clone was constructed by co-ligating an EagI fragment from H36N03 with an EagI–EcoR1 fragment from H35P10 into the EagI–EcoR1 site of pBSIIKS(+). The pPK348 plasmid was derived by inserting the *gfp* cassette from pPD103.87 flanked by XbaI ends into the unique SpeI site present in pPK347. Co-injection of 10 ng/μl pPK348 and 80 ng/μl pRF4 led to the generation of the transgenic array *crEx40* (Mello and Fire, 1995). The *ptc-3*(*ok121*)*; crEx40* strain was generated by mating *ptc-3*(*ok121*)*/sup-9*; *unc-93* rubber band hermaphrodites with males carrying *crEx40*. The genotype of *ptc-3*(*ok121*)*; crEx40* animals (strain PK173) was verified by performing single-worm PCR, as described above. Plasmid pPK688 (*ptc-3::mRFP*) was generated by ligating an in-frame mRFP cassette with SpeI ends from pPK687 into the unique SpeI site of pPK347 (described above). DNA sequencing revealed that pPK688 carried two in-frame copies of mRFP.

Oligonucleotide sequences:

SL1 5′ GGTTTAATTACCCAAGTTTGAG 3′
SL2 5′ GGTTTTAACCCAGTTACTCAAG 3′
ptc-3_311F 5′ GCCGAGTTGGCTGGCTACG 3′
ptc-3_262R 5′ CCGACATTTCGACCTCCG 3′
ptc-3_a1 5′ atcggatccCTGACAAAGTTTAACGAGTGG 3′
ptc-3_a2 5′ atcgaattcCACATCTCCATAGTTCGGGTA 3′
ptc-3_5c 5′ GAACACACAACCCGTTGAAC 3′
ptc-3_6c 5′ AGTGGCTGCCCATGGATTG 3′
ptc-3_12c 5′ CTCCGACGGTGTACCACG 3′
ptc-3_14c 5′ CTCAGATCGATTCCGATCATTGC 3′
ptc-3_15c 5′ GAGAACGTATCCCGAGATGAAG 3′
ptc-3_23c 5′ CCTTCTGTGCCTTGAGTACGG 3′
ptc-3_24c 5′ TGGAGATGTGATGACCGGTG 3′
lin-48_MluF1 5′ atcacgcgtCCTGCATTTTTTTCAGAGTC 3′
lin-48_NheR1 5′ atcgctagcCTGAAATTGAGCAGAGCTGAA 3′
let-60_MluF1 5′ atcacgcgtCAGTCAGTAGAATACAAAATTTTAG 3′
let-60_NheR1 5′ atcgctagcTACCCTTTTCTGAAAAAAGACG 3′
dpy-7p_F1 5′ tgctagcCTCATTCCACGATTTCTCGC 3′
dpy-7p_R1 5′ tgctagcTCTGGAACAAAATGTAAGAATATTC 3′
ptc-3_NheF1 5′ atcgctagcATGAAGGTGCATTCGGAACAAC 3′
ptc-3_XbaR1 5′ atctctagaccgcggttaaCAAAAGCTGGGTACCGGGC 3′

### Tissue-specific and intracellular expression

The promoter regions from genes *lin-48* (Wang and Chamberlin, 2002), *let-60* (Dent and Han, 1998), and *dpy-7* (Gilleard et al., 1997; Myers and Greenwald, 2005) were amplified from wild-type N2 genomic DNA using the appropriate primer pairs (above). These primers carry restriction sites suitable for ligating the PCR amplified promoter region to a *ptc-3::gfp* expression cassette, which was PCR amplified from pPK348 using the primers ptc-3_NheF1 and ptc-3_XbaR1. The following plasmids were generated after ligating the *ptc-3::gfp* expression cassette with the designated promoter sequence: pPK699 (*lin-48p::ptc-3::gfp*), pPK700 (*let-60p::ptc-3::gfp*), and pPK720 (*dpy-7p::ptc-3::gfp*). Plasmid sequences were verified by DNA sequencing (Eurofins). Transgenic arrays, which are listed in brackets, were generated by co-injecting 10 ng/μl pPK699 (*crEx345, crEx440, crEx441*) or pPK700 (*crEx348*, *crEx442, crEx443*), with either 100 ng/μl pRF4 or 50 ng/μl pTG96. The *crEx437* and *crEx438* extrachromosomal arrays (*dpy-7p::ptc-3::gfp*) were generated by co-injecting 5 ng/μl pPK720 with 50 ng/μl of p*unc-122::mRFP* and 50 ng/μl pBSIIKS(+). The following strains were established by standard genetic methods: PK2894 [*ptc-3(ok121); crEx437*] and PK2937 [*ptc-3(ok121)*; *crEx442*].

Strain PK2766, which carries the extrachromosomal array, *crEx341* (*ptc-3::mRFP*), was established by co-injecting 5 ng/μl pPK688 and 50 ng/μl pTG96 [(NLS) *sur-5::GFP*]. Strain PK2770, *ptc-3(ok121)*; *crEx341*; *jcIS1* [*ajm-1::gfp;* pRF4] was constructed by standard genetic methods.

### Expression analysis

Nomarski DIC and fluorescent images were captured using Improvision Open Lab software (v. 4, Perkin Elmer) and a Zeiss Axioskop equipped with an ORCA ER digital CCD camera (Hamamatsu Photonics K.K.). Images were also acquired using Image-Pro Plus 7.0 (MediaCybernetics) and an EXi Aqua camera (QImaging). Confocal images were obtained using a Perkin Elmer UltraVIEW spinning disk system attached to a Leica DMI6000 inverted microscope controlled by Volocity (Perkin Elmer) software and captured using a Hamamatsu EMCCD camera. Images were post-processed for publication using Adobe Photoshop CS 4.

### Site-directed mutagenesis

Site-directed mutagenesis (SDM) was used to convert the DNA sequence encoding the PTC-3 GxxxD motif associated with the permease transporter domain to GxxxA and to disrupt the SSD D(780)N. Briefly, a 1.762 kb Pml I DNA fragment from pPK348 was sub-cloned into pBSIIKS(+) and subjected to 25 cycles of PCR using *PfuUltra* HF DNA polymerase (Stratagene) and complementary oligonucleotides carrying the desired base changes (see below), before being digested with DpnI and transfected into calcium competent XL-1 Blue bacteria. The modified PmlI fragment was used to replace the homologous sequence in pPK348. The SDM changes in the resulting clones, pPK660 [SSD, D(780 N)] and pPK674 [GxxxD, D(697)A] were verified by DNA sequencing (Eurofins), as were the transgenes produced using these clones.

GxxxA(+) 5′ CTTTCCTTAGGTCTAGGAATCGcTGATATGTTCCTCCTTCTGC
GxxxA(−) 5′ GCAGAAGGAGGAACATATCAgCGATTCCTAGACCTAAGGAAAG
D(780)N (+) 5′ CCAGCAATGATCGGAATCaATCTGAGAAGACAGCGAAAAG
D(780)N (−) 5′ CTTTTCGCTGTCTTCTCAGATtGATTCCGATCATTGCTGG

## Results

### Molecular identification and characterization of *ptc-3*

**Fig. 1:**
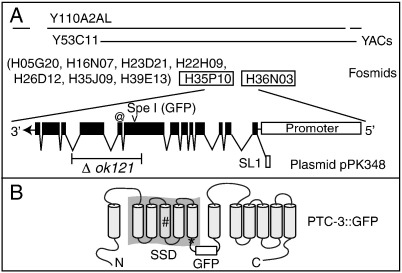
Characterization and cloning of the *C. elegans ptc-3* locus. (A) YAC and fosmid clones carrying *ptc-3* genomic sequences. The plasmid pPK348, which rescues a *ptc-3* deletion mutant, was constructed from genomic sequences contained on fosmids; a *gfp* expression cassette was inserted at the SpeI restriction site in exon 8. The putative promoter and intron/exon organization of *ptc-3* is displayed (5′-3′, right to left); the extent of the *ptc-3*(*ok121*) deletion is indicated. The position of a 9 bp sequence, which is present in the *ptc-3b* cDNA at the 5′ end of exon 9, but not in the *ptc-3a* cDNA, is indicated (@). (B) Topological arrangement of the PTC-3 protein highlighting the SSD region. The positions of the SSD D(780)N missense change (*) and the D(697)A missense change (#) in the GxxxD/E motif associated with the permease transporter domain are indicated.

The *C. elegans ptc-3* gene was first identified when the cDNA clone yk68c12 (kind gift of Y. Kohara) was found to encode a peptide that shared similarity, but not identity with PTC-1. Subsequently it was found that the *ptc-3* gene lies in a cosmid gap, so we cloned the gene locus using fosmids, which were identified by hybridizing a fosmid grid (kind gift of R. Shownkeen) with a probe generated from the yk68c12 cDNA sequence (Fig. 1A).

The coding potential of *ptc-3* was determined by sequencing multiple *ptc-3* cDNA clones. Taken together, our data indicate that the *ptc-3* locus produces two alternative isoforms, *ptc-3a* and *ptc-3b*, which differ by only nine nucleotides at the beginning of exon 9. These isoforms potentially encode proteins of 1358 and 1361 amino acids, respectively (Fig. 1A). RT-PCR also revealed that both mRNAs are trans spliced to SL1. BLAST analysis indicates that the putative PTC-3 protein shares the greatest similarity with the *C. elegans* PTC-1 protein (46% identity, 64% similarity), and that it also shares ~ 34% identity with *Drosophila* and vertebrate Ptc2 proteins. Hydropathy analysis indicates that PTC-3 has 12 potential membrane spanning domains, which share a topological arrangement similar to that predicted for other PTC proteins (Fig. 1B).

### *ptc-3* is an essential gene but displays a reduction-of-function phenotype that is distinct from *ptc-1*

We previously showed that *ptc-1* is an essential gene involved in germ line cytokinesis (Kuwabara et al., 2000); the absence of *ptc-1* resulted in sterility due to the formation of multinucleate germ cells. To determine if the activity of *ptc-3* resembled that of *ptc-1*, *ptc-3*(*RNAi*) was performed by microinjecting *ptc-3* double-stranded (ds) RNA into the germ line syncytium of adult hermaphrodites. An obvious germ line cytokinesis was not observed in either the *ptc-3*(*RNAi*) treated parental animals or in F_1_ progeny, which survived to adulthood. However, *ptc-3*(*RNAi*) resulted in a high level of embryonic and larval lethality (Table 1).

### Characterization of *ptc-3* deletion mutant

To establish the likely null phenotype of *ptc-3* and to elucidate its role in development, we obtained a *ptc-3*(*ok121*) deletion mutant from the *C. elegans* Knockout Consortium and outcrossed it five times prior to analysis. We then sequenced a PCR product spanning the deletion and showed that the *ok121* allele removes 1.993 kb of *ptc-3* DNA sequence between exon 8 and intron 10 (Fig. 1A); hence, the absence of an extensive region of coding sequence suggests that *ptc-3*(*ok128*) is likely to be null.

Due to the paucity of genetic balancers in the vicinity of *ptc-3*, the *ptc-3(ok121*) mutation was initially maintained by generating a partially balanced *ptc-3*(*ok121*)*/sup-9*(*n180*); *unc-93*(*n1500sd*) strain. However, owing to the marginal fertility of the balanced *ptc-3* strain, the phenotype of homozygous *ptc-3* mutants was examined by analyzing the progeny segregating from a *ptc-3*(*ok121*)/+ heterozygous mother; single-worm PCR was used to establish the presence of the *ptc-3* deletion allele. We found that *ptc-3*(*ok121*)/+ hermaphrodites produced an average of 59.7 ± 11.2 dead eggs and 210.5 ± 37.5 viable progeny (*n* = 15), which developed into fertile adults (Table 1). Our inability to recover any viable *ptc-3* homozygotes indicates that the absence of *ptc-3* leads to embryonic lethality.

**Fig. 2:**
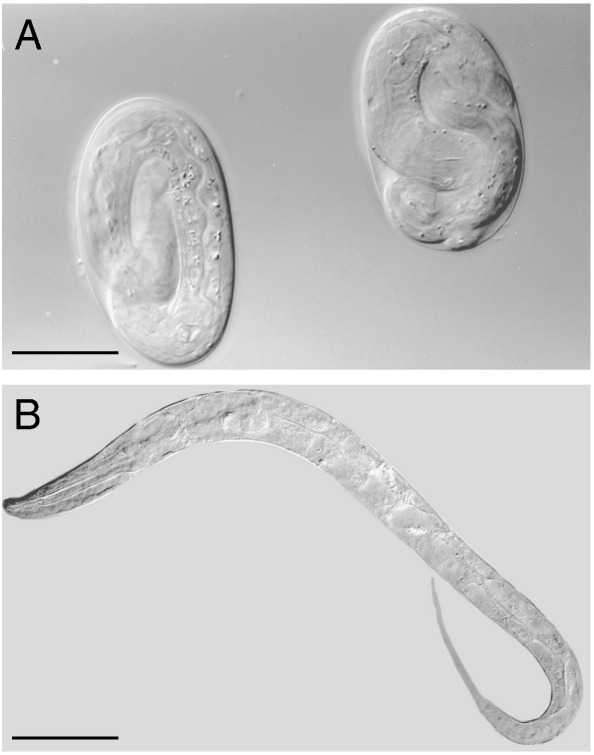
*ptc-3*(*ok121*) causes recessive embryonic lethality. (A) Nomarski DIC micrograph of *ptc-3* late-stage embryos prior to hatching. (B) The embryo shown in panel (A), right, was photographed immediately after hatching when it developed extensive vacuolation and died. Scale bars, 20 μm.

Phenotypic inspection revealed that *ptc-3* embryos died around the time of hatching. Prior to hatching, embryos displayed essentially normal morphogenesis and muscle activity with vigorous tumbling within the eggshell (Fig. 2A); however, when embryos hatched out of the protective barrier provided by the eggshell into L1 stage larva, they died with a fluid-filled appearance. The developmental stage when death occurred and the fluid-filled appearance of the dead animals, such as the individual shown in Fig. 2B, indicate that the absence of *ptc-3* results in an apparent defect in osmoregulation. Hence, both *ptc-1* and *ptc-3* play essential roles in *C. elegans*, but each displays a distinct reduction-of-function phenotype.

### Postembryonic roles for *ptc-3* revealed by RNAi

Preliminary *ptc-3*(*RNAi*) feeding studies provided hints that *ptc-3* is required during postembryonic development (Zugasti et al., 2005). Given that the absence of *ptc-3* leads to embryonic lethality, additional RNAi feeding experiments were performed using populations of synchronized L1 larvae in order to bypass the critical period when *ptc-3* is known to be required for embryonic viability.

**Fig. 3:**
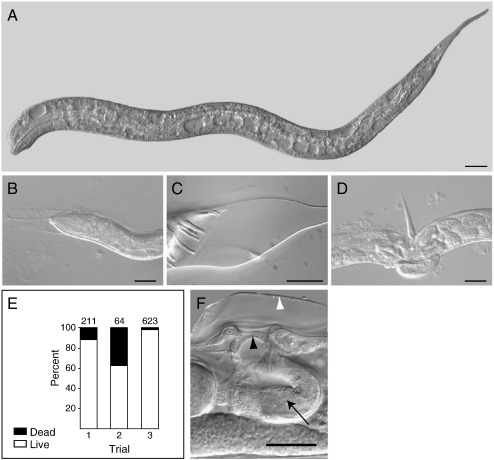
*ptc-3*(*RNAi*) leads to lethality and to postembryonic morphological defects. (A) L2 stage *ptc-3*(*RNAi*) larva showing lethal vacuolation. (B–D) *ptc-3*(*RNAi*) causes variable defects in molting. (E) Attenuated *ptc-3*(*RNAi*) trials initiated using synchronized L1 larvae result in less lethality. Numbers of animals involved in each trial are indicated above the bar. White fill, viable animals; black fill, dead animals. (F) Egl animal with defective vulval morphogenesis. Black arrowhead, thickened hymen; arrow, L1 stage larva trapped within the uterus; white arrowhead, unshed cuticle covering vulval opening. Lateral view, ventral side up. Scale bars, 20 μm.

The worms most severely affected by *ptc-3*(*RNAi*) arrested during larval development and became unresponsive to touch by the L4/adult stage. Worms that survived to adulthood were usually shorter than wild-type animals and died without producing viable progeny. When the dead and dying worms were examined in more detail, it was apparent that most displayed extensive vacuolation (Fig. 3A). Larvae encased in unshed cuticle were also observed, indicating that the absence of *ptc-3* disrupts molting (Fig. 3B–D); larvae trapped within unshed cuticle were also unable to obtain food (Fig. 3B). Male-specific *ptc-3*(*RNAi*) molting defects were also observed, which prevented the unfurling of the mature tail rays (Fig. 3C). Taken together, these defects indicated that there is a postembryonic requirement for *ptc-3* activity.

In experiments involving a deliberate attempt to weaken the effect of *ptc-3*(*RNAi*) (Fig. 3E), a consistent range of specific, but less penetrant and hence more difficult to quantify, morphological defects were also detected. The primary morphological defect under these conditions was a defect in egg laying (Egl); the most severely affected Egl animals, despite being well fed, contained L1 larvae within the uterus. Molting defects, some of which left cuticle constrictions encircling the vulval region, were also observed (Fig. 3D). Other Egl animals displayed defects in vulval morphogenesis, which produced protruding vulvae (Pvl) or in very rare instances vulvaless (Vul) animals. In a number of cases, severely Egl animals were found to have a thickened hymen, which prevented eggs from being expelled from the uterus (Fig. 3F). The observation that *ptc-3*(*RNAi*) treated animals often ruptured through the vulva during microscopic examination when gentle pressure was applied from a cover slip provided further evidence that a reduction of *ptc-3* activity compromised vulval development. Adult *ptc-3*(*RNAi*) survivors with under-proliferated germ lines were also identified. However, the multinucleated germ cells present in *ptc-1* mutants were never observed in *ptc-3* mutants.

### Transgenic rescue of *ptc-3*(*ok121*) and dynamic expression of a *ptc-3::gfp* translational reporter

We next constructed plasmid pPK348, which carries the entire predicted *ptc-3* locus comprised of a 6.9 kb genomic coding region fused in-frame to GFP and more than 5 kb of 5′ upstream sequence, including the putative promoter (Fig. 1B). This plasmid was used to generate *crEx40* transgenic animals, which express *ptc-3p::ptc-3::gfp*. To minimize any potential disruption to the activity of the PTC-3 protein, which has 12 potential membrane spanning domains, the GFP expression cassette was inserted within a predicted cytoplasmic loop that separates two sets of 1 + 5 TM domains (Fig. 1B). The design of this construct was based on the observation that Ptc activity could be reconstituted in a manner similar to that displayed by bacterial transporters when the two 1 + 5 TM domain cassettes of *Drosophila* Ptc were physically separated before being co-expressed in *Drosophila* (Johnson et al., 2000). We found that *crEx40* fully rescued the lethality of *ptc-3*(*ok121*) mutants, based on the ability to detect fertile transgenic *ptc-3* homozygous adults. This indicates that the PTC-3::GFP protein retained function despite the presence of GFP (Table 1). As might be expected, all of the surviving *ptc-3*(*ok121*); *crEx40* animals displayed an obligate Roller phenotype resulting from the expression of the co-transformation marker *rol-6*(*d*). Moreover, the transgenerational survival of *ptc-3*(*ok121*) homozygotes was dependent on retention of *crEx40*, indicating that the phenotypes attributed to the deletion of the *ptc-3* gene were not caused by a linked background mutation. Clonal PCR analysis of single worms verified that the *crEx40* array was present in a *ptc-3* mutant background. In an attempt to generate *ptc-3* genetic mosaics, 450 L4 *ptc-3; crEx40* hermaphrodites were individually plated and the phenotypes of their progeny examined. We identified three animals that had presumably lost the *crEx40* array in the germ line lineage because they produced only dead eggs (178 ± 27). The existence of these mosaics provided further support that *ptc-3* is an essential gene.

**Fig. 4:**
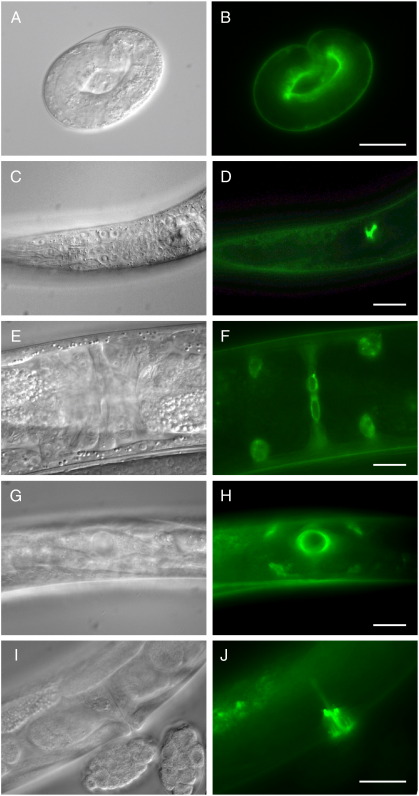
Expression of a *ptc-3::gfp* translational fusion in homozygous *ptc-3; crEx40* hermaphrodites. (A, B) Embryonic hypodermal expression prior to hatching. (C, D) L1 stage expression in the excretory duct. (E, F) Expression in the uterine utse cell, ventral view. (G, H) Expression in the toroidal vulD and vulE cells of the vulva. The edges of the utse cell can also be discerned. (I, J) Expression in the vulval VC4/VC5 neuron. Images on left are DIC Nomarski micrographs, images on the right are the same as those on the left illuminated with epi-fluorescence to visualize GFP. Scale bars, 20 μm.

The analysis of *ptc-3*; *crEx40* animals revealed that transgenic PTC-3::GFP expression is highly dynamic (Fig. 4). In particular, high levels of PTC-3::GFP were detected in the hypodermis prior to hatching; expression also intensified in the hypodermis prior to each larval molt (Fig. 4A, B; data not shown). PTC-3::GFP was also detected in the excretory duct (Fig. 4C, D), which is involved in maintaining ionic and osmotic regulation (Nelson and Riddle, 1984); *ptc-3p::ptc-3::gfp* expression in the excretory duct is of potential relevance in explaining the lethality of *ptc-3* mutants. During the L3 and L4 larval stages, PTC-3::GFP was detected during the morphogenesis of the somatic gonad and vulva. Prominent expression was associated with the H-shaped uterine seam cell (utse), which connects the uterus and vulva (Fig. 4E–H). During vulval morphogenesis, PTC-3::GFP was also associated with the toroidal vulE, and possibly vulD cells (Fig. 4G, H). In addition, expression was detected in the VC4/VC5 neurons of the vulva (Fig. 4I, J).

**Fig. 5:**
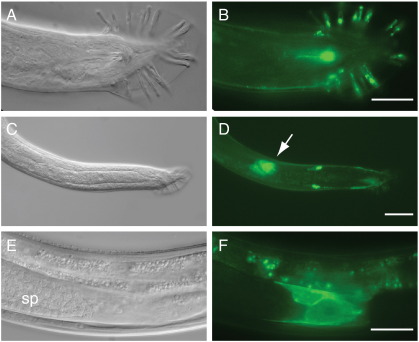
Expression of a *ptc-3::gfp* translational fusion in adult XO *ptc-3* homozygous males. (A, B) The reporter is expressed in rays of the male tail. (C, D) Arrow indicates expression in cells of the somatic gonad between the vas deferens and seminal vesicle; a pair of neurons also expresses the reporter. (E, F) Enlarged view showing PTC-3 expression in the valve region separating the seminal vesicle and vas deferens of the somatic gonad. sp, spermatids. Scale bars, 20 μm.

Expression of *ptc-3p::ptc-3::gfp* was also examined during the L4 and adult stages of XO males (Fig. 5). In males, PTC-3::GFP was associated with the precursor and mature sensory rays, the cloaca, and pre-anal ganglia and cephalic neurons (Fig. 5A, B). In addition, PTC-3::GFP was expressed in five cells found in the valve region between the seminal vesicle and vas deferens of the somatic gonad (Fig. 5C–F).

### *lin-48p::ptc-3* expression in the excretory duct cell fails to rescue *ptc-3* lethality

**Fig. 6:**
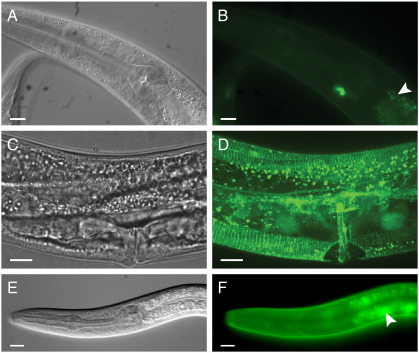
*ptc-3::gfp* expression driven from the *lin-48*, *let-60* or *dpy-7* promoter. (A, B) *lin-48p::ptc-3::gfp* expression in the excretory duct. (C, D) *let-60p::ptc-3::gfp* is expressed in multiple tissues. (D) Merged Z-stack of 42 sections, each with a thickness of 0.5 μm. (E, F) *dpy-7p::ptc-3::gfp* is expressed in hypodermal cells. Images on left are DIC Nomarski micrographs, images on the right are the same as those on the left illuminated with epi-fluorescence (excluding D) to visualize GFP. Arrowhead, autofluorescence from gut. Anterior, left; ventral, down. Scale bars, 10 μm.

To further examine the requirement for *ptc-3* activity during development, we sought to identify the tissue(s) requiring *ptc-3* activity. Given that *ptc-3p::ptc-3::gfp* expression is detected in the excretory duct and that the fluid-filled phenotype of *ptc-3* mutants could arise from a defect in excretory duct function, we first attempted to rescue *ptc-3* mutants with a *lin-48p::ptc-3::gfp* reporter. We selected the *lin-48* promoter for this purpose because it was previously shown that *lin-48* is expressed in the excretory duct, hindgut cells, and several neuronal support cells (Wang and Chamberlin, 2002). We generated three *lin-48p::ptc-3::gfp* transgenic lines (*crEx345, crEx440* and *crEx441*) and found that despite showing the expected *lin-48* pattern of expression in the excretory duct cell (Fig. 6A, B) hindgut and neuronal support cells (data not shown), none of the arrays rescued the lethality of *ptc-3* homozygotes. Thus, it would appear that expression of *ptc-3* in the subset of cells associated with *lin-48* activity is not sufficient to rescue the lethality of the *ptc-3* mutation.

### Hypodermal expression of *ptc-3* is crucial for activity

Because the *lin-48p::ptc-3::gfp* reporter failed to rescue *ptc-3* mutants, we attempted to drive *ptc-3::gfp* expression from the *let-60* Ras promoter, which is expressed in multiple tissues, including the vulval precursor cells and hypodermis (Dent and Han, 1998). We generated three transgenic *let-60p::ptc-3::gfp* lines (*crEx348*, *crEx442*, and *crEx443*) and selected *crEx442* for further study because of its strong GFP expression. Similar to the pattern reported by Dent and Han (1998), we observed that the *crEx442 let-60p::ptc-3::gfp* reporter is broadly expressed in multiple tissues (Fig. 6C, D). Hermaphrodites carrying the *crEx442* array were also found to be severely Egl (egg laying defective); however, it is unknown whether ectopic expression of *ptc-3* in vulval cells or squelching of the native *let-60* promoter was responsible for this phenotype. By contrast to the result obtained using the *lin-48p::ptc-3::gfp* reporter, we found that *ptc-3*; *crEx442* animals developed into fertile adults capable of transgenerational survival (Table 1).

Because both the *ptc-3p::ptc-3::gfp* and *let-60p::ptc-3::gfp* reporters are expressed in the hypodermis, we next sought to determine if the hypodermis is the principle site of *ptc-3* activity by driving expression using the *dpy-7* promoter. The *dpy-7* gene encodes a cuticular collagen that is expressed in P cells, hyp7 and most other hypodermal cells of the head and tail, but not in the VPCs (Gilleard et al., 1997; Myers and Greenwald, 2005). To test the activity of the *dpy-7p::ptc-3::gfp* reporter, we generated the *crEx437* extrachromosomal array, which also carries the *unc-122::mRFP* coelomocyte marker, and found that hypodermal *dpy-7p::ptc-3::gfp* expression (Fig. 6E, F) was indeed sufficient to rescue the *ptc-3* mutant phenotype (Table 1). Similar to the results obtained with both *crEx40* and *crEx442*, we found that transgenerational survival of *ptc-3* homozygotes was dependent on the retention of the *crEx437* array. However, unlike the rescue achieved with the native *ptc-3* promoter driving *ptc-3::gfp*, 3% of the *let-60p::ptc-3::gfp* and 5% of the *dpy-7p::ptc-3::gfp* animals died as larvae with a fluid-filled appearance; this low level of lethality could arise either as a result of partial promoter insufficiency or from the somatic loss of the array (Table 1).

### Intracellular localization of PTC-3::GFP

**Fig. 7:**
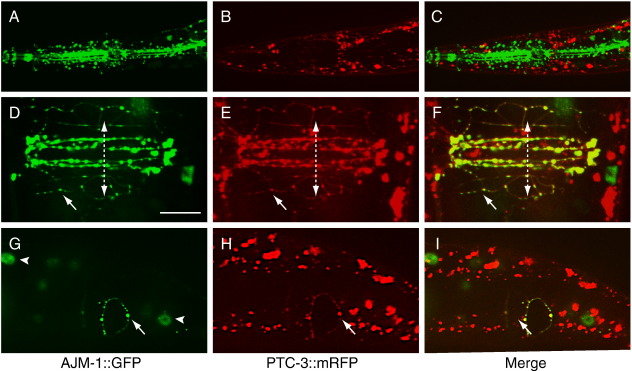
Co-localization of AJM-1::GFP and PTC-3::mRFP on the apical surface of hypodermal cells and vulval toroids in *ptc-3(ok121)*; *jcIS1 [ajm-1::gfp]; crEx341* [*ptc-3::mRFP + sur-5::gfp*] hermaphrodites. (A–C) AJM-1::GFP and PTC-3::mRFP fail to co-localize at pharyngeal adherens junctions. Merged Z-stack of 42 sections, each with a thickness of 0.5 μm. (D–F) Ventral view showing AJM-1::GFP and PTC-3::mRFP expression in vulval toroids and adjacent hypodermal cells. Dashed line with arrowheads indicates the direction of the ventral midline. Merged Z-stack of 8 sections, each with a thickness of 0.5 μm. (G–I) Co-localization of AJM-1::GFP and PTC-3::mRFP in posterior hypodermal cells, with posterior right. Merged Z-stack of 3 sections, each with a thickness of 0.5 μm. Arrows, hypodermal puncta co-expressing AJM-1::GFP and PTC-3::mRFP; arrowheads, nuclei expressing the SUR-5::GFP co-transformation marker. Scale bar, 10 μM.

Expression of the *ptc-3p::ptc-3::gfp* reporter is predominantly associated with polarized epithelial cells at the plasma membrane and in vesicular structures. To determine whether PTC-3 is enriched on the apical or basolateral surface, we first generated the extrachromosomal array *crEx341*, which expresses *ptc-3::mRFP* and the nuclear co-transformation marker *sur-5::gfp*. We then demonstrated that *crEx341* was capable of rescuing a *ptc-3* mutant; the survival of homozygous *ptc-3* animals was dependent on retention of the *crEx341* extrachromosomal (data not shown). Subsequently, we constructed the strain *ptc-3(ok121); jcIS1* [*ajm-1::gfp*]; *crEx341*, which co-expresses *ptc-3::mRFP*, the adherens junction marker *ajm-1::gfp* and the nuclear marker *sur-5::gfp*. Confocal microscopy revealed that the expression patterns of *ptc-3::mRFP* and *ajm-1::GFP* were not identical, as demonstrated for the pharyngeal region (e.g., Fig. 7A–C); however, in cells in which the two proteins were co-expressed, such as the hyp cells and the vulval toroids, the two reporters displayed extensive co-localization at the apical surface (Fig. 7D–I).

### PTC-3 activity requires an intact transporter domain, but not an SSD

The PTC-3 protein shares sequence similarity with proteins that carry a SSD and permease transporter domain, including prokaryotic proteins that are members of the RND superfamily of efflux permeases (Tseng et al., 1999). To examine the requirement for the SSD in PTC-3 function, we generated a Clustal W multisequence alignment of SSDs to identify the position of an invariant Asp, which when modified to Asn in the SREBP-cleavage activating protein (SCAP) confers cholesterol resistance in mammalian cells (Suppl. Fig. 1) (Hua et al., 1996). In *Drosophila*, the replacement of a homologous Asp residue renders Ptc unable to repress Smo (Martin et al., 2001; Strutt et al., 2001).

To ask whether the replacement of this invariant Asp affected PTC-3 activity, we engineered a base-change in the pPK348 plasmid to construct a PTC-3(SSD, D780N)::GFP reporter (Fig. 1B). Three independent transgenic PTC-3(SSD, D780N)::GFP lines (*crEx232*, *crEx233* and *crEx*234) were generated and each was tested and found to rescue the lethality of *ptc-3* homozygotes; all survivors displayed an obligate roller phenotype indicative of array retention. Table 1 demonstrates the ability of *crEx234* to rescue *ptc-3* homozygotes. Analysis of *ptc-3; crEx234* homozygotes further revealed that the pattern of PTC-3(SSD, D780N)::GFP expression was virtually indistinguishable from that of the comparable wild-type transgene (Suppl. Fig. 2). The possibility that the rescuing activity was provided by a rare recombination event between the deletion allele and the transgene was eliminated because Asp(780) of the SSD is deleted in *ptc-3*(*ok121*).

We next asked whether the sequence similarity shared between PTC-3 and RND transporters could provide insights into *ptc-3* activity. The prokaryotic RND efflux pumps and PTC proteins share a similar internally duplicated 1 + 5 arrangement of TM domains (Saier, 1994; Tseng et al., 1999). In prokaryotic RND transporters, the GxxxD/E motif associated with permease transporters is found in the middle of TM4 and is essential for pump activity (Goldberg et al., 1999; Guan and Nakae, 2001). PTC-3 carries an extended GxxxD(D) motif in TM 4 and a GxxxE motif in TM 10; TM 4 and 10 are equivalently positioned based on the 1 + 5 convention of TM numbering (Suppl. Fig. 3). Site-directed mutagenesis of plasmid pPK348 was used to modify the GxxxD motif in TM 4 and to create a PTC-3(GxxxD, D697A)::GFP reporter (Fig. 1B). Three independent PTC-3(GxxxD, D697A)::GFP transgenic lines were established (*crEx235*–*crEx237*), but none rescued the lethality of *ptc-3*(*ok121*) homozygotes. Clonal analysis revealed that *ptc-3*(*ok121*) mutants died as embryos despite carrying a PTC-3(GxxxD, D697A)::GFP reporter transgene. We were unable to detect any differences in the expression pattern of the PTC-3(GxxxD, D697A)::GFP when compared to the PTC-3::GFP or PTC-3::GFP (SSD, D780N) reporters (Suppl. Fig. 4). Hence, disruption of the permease transporter domain interferes with PTC-3 activity but does not obviously affect the pattern of PTC-3 expression. Taken together, our analysis indicates that an intact GxxxD motif in TM 4, but not the SSD, is required for *ptc-3* activity.

## Discussion

Members of the large *C. elegans* family of PTC and PTR proteins share extensive sequence similarity with *Drosophila* and vertebrate Ptc proteins, which function as membrane receptors for Hh (Zugasti et al., 2005). Within the *C. elegans* family of proteins, PTC-1 and PTC-3 are the homologs most closely related by sequence to *Drosophila* and vertebrate Ptc proteins. Most of the *C. elegans ptc* and *ptr* genes display reduction-of-function phenotypes, which variously include defects in cytokinesis, molting, endocytosis, body morphology, and lumen formation (Kuwabara et al., 2000; Zugasti et al., 2005; Perens and Shaham, 2005); however, only a small number of these genes appear to be essential. Here we report that *ptc-3* is one such gene. The absence of *ptc-3* leads to lethality at the transition between embryonic and larval development, a phenotype that is distinct from that displayed by *ptc-1* in germ line cytokinesis. The dynamic expression pattern of a rescuing *ptc-3p::ptc-3::gfp* reporter in *ptc-3* homozygotes further suggested that *ptc-3* might have additional roles in postembryonic development. In support of this hypothesis, *ptc-3*(*RNAi*) treated animals displayed larval lethality, incomplete molting, decreased body size, and disrupted vulval morphogenesis indicating that *ptc-3* activity is required throughout larval development. The finding that many members of the *ptr* family display a similar range of RNAi phenotypes raises the possibility that *ptc-3* might also function in the same processes or pathways as a subset of these *ptr* genes (Zugasti et al., 2005).

### An intact SSD is not essential for PTC-3 function

The PTC, PTR, Disp, and NPC1 proteins share sequence similarity and carry a SSD and RND permease domain. To understand how the *C. elegans* PTC-3 protein affects *C. elegans* development in the absence of Hh and Smo, we examined the importance of the SSD and RND permease domain to *ptc-3* activity.

Proteins that carry an SSD are generally involved in sterol metabolism or vesicle transport (Kuwabara and Labouesse, 2002; Eaton, 2008). The importance of the SSD is exemplified by its role in SCAP, a protein that shuttles between the endoplasmic reticulum (ER) and Golgi in response to sterol levels. SCAP is retained in the ER by an ER-resident protein, but under conditions of low cholesterol, SCAP mediates the transport of the sterol regulatory element binding protein (SREBP) from the ER to the Golgi where it is proteolytically processed to generate an active transcription factor. In mammals, SCAP is rendered insensitive to regulation by sterols in cultured cells by the SSD D(443)N mutation (Hua et al., 1996). Mutations in the SSD also inactivate and disrupt the intracellular transport of cholesterol by NPC1 (Watari et al., 1999). In the SCAP protein, the affected Asp residue is located at an intracellular membrane-spanning boundary of the SSD and is highly conserved across members of the family. The homologous D(584)N replacement in the *Drosophila* Ptc protein results in dominant negative activity (Martin et al., 2001; Strutt et al., 2001; Johnson et al., 2002). In mouse and human cultured cells, the corresponding mutations in Ptc(D585N) and PTCH(D585N) had no appreciable effect on their activities (Johnson et al., 2002; Taipale et al., 2002). Similarly, the *C. elegans* DAF-6/PTR-7 protein also retained function when the SSD region was disrupted, although a fraction of protein appeared to be mis-localized (Perens and Shaham, 2005).

In our hands, replacement of the conserved Asp residue in the PTC-3(D780N) SSD did not disrupt Ce-PTC-3 activity; transgenic expression of PTC-3(D780N) rescued the late embryonic lethality of *ptc-3* mutants and supported virtually normal development. Moreover, the expression pattern of PTC-3(D780N)::GFP was indistinguishable from that observed for the wild-type reporter. However, it remains possible that a modest reduction in PTC-3(D780N) activity could be compensated by (increased) transgenic expression or perhaps by the potentially overlapping activity of one or more of the PTR proteins (Zugasti et al., 2005). Taken together, our results support observations indicating that mutations in the SSD of Ptc can have different effects on family members, although the ability to measure such effects could be sensitive to expression levels.

### Disruption of the GxxxD/E motif associated with the permease transporter domain abolishes PTC-3 activity

When we aligned the PTC and PTR proteins sequences, it became apparent that only PTC-1, PTC-3, and five of the 24 PTR proteins have GxxxD/E motifs in both TM 4 and TM 10 (Suppl. Fig. 3). In RND permease transporters, the aspartic acid residue in the GxxxD/E motif is associated with the proton binding and antiporter activity of bacterial efflux pumps (Goldberg et al., 1999; Guan and Nakae, 2001). The only *C. elegans* PTC family protein for which the role of the RND permease domain has been investigated so far is DAF-6/PTR-7, which is proposed to participate in lumen formation (Perens and Shaham, 2005). However, unlike the *Drosophila*, vertebrate and worm Ptc proteins, DAF-6/PTR-7 does not have a GxxxD/E motif in TM 4 and disruption of a motif in TM10 had no significant effect on DAF-6 function. Thus, it was concluded that permease activity was not required for DAF-6/PTR-7 activity although it had a minor effect on protein localization.

By contrast, replacement of the invariant Asp (697) with Ala in the permease transporter GxxxD/E motif in TM 4 was sufficient to impair PTC-3 activity, although the pattern of PTC-3(GxxxD, D697A)::GFP expression remained similar to that of the wild-type reporter. In humans, missense mutations in the conserved residues of the permease transporter GxxxD/E motif have also been detected in Gorlin's syndrome, a hereditary condition caused by familial mutations in *PTCH* (Chidambaram et al., 1996; Wicking et al., 1997; Aszterbaum et al., 1998). Therefore, a dependence on the permease transporter domain for protein activity has been observed for both the *C. elegans* and human Ptc proteins. It is interesting to speculate that when the *C. elegans patched* gene family expanded to generate the *ptc-related* genes, the dependence on the permease transporter domain for activity was lost in some *ptr* members, such as *daf-6/ptr-7*. This difference could help to explain why DAF-6 does not require an intact GxxxD/E motif. A requirement for permease transporter activity has also been demonstrated for Disp and the cholesterol transporter Niemann-Pick C1 (NPC1) proteins (Davies et al., 2000; Ma et al., 2002).

Is it possible that Ptc proteins have transporter activity? The mechanism by which Ptc inhibits Smo has long been a puzzle. Early models suggested that Ptc bound and stabilized Smo in an inactive state; however, a definitive physical interaction between the two proteins has never been convincingly demonstrated (Denef et al., 2000; Taipale et al., 2002). An important insight was obtained when Ptc was shown to act catalytically at substoichiometric levels to inhibit Smo activity (Taipale et al., 2002), an observation that led to the hypothesis that Ptc might transport a small molecule capable of repressing Smo. Multiple lines of evidence now indicate that Smo is regulated by an endogenous small molecule, possibly a sterol derivative (Chen et al., 2002a, 2002b; Frank-Kamenetsky et al., 2002); specific oxysterols have also been found to be potent activators of Hh signalling mediated through Smo (Corcoran and Scott, 2006; Dwyer et al., 2007). It has also been reported that (Pro) vitamin D3, in the form of 7-DHC (dehydrocholesterol) or its metabolite vitamin D3, can be pumped out of cells in culture by Ptc and repress Smo activity in a cell non-autonomous manner (Bijlsma et al., 2006). Furthermore, vitamin D3-treated zebrafish embryos phenotypically resemble a *smo* ^−/−^ mutant. However, it remains unclear how vitamin D3 impinges on this pathway, because this study does not fully accommodate the observation that Ptc regulates Smo autonomously, nor does it explain how Ptc activity can be driven by receptor occupancy (Casali and Struhl, 2004). More recently, Ptc has been implicated as playing a role in suppressing the accumulation of phosphatidylinositol-4 phosphate (PI4P), which in turn, leads to Smo repression (Yavari et al., 2010).

How do we explain the role of *ptc-3* in *C. elegans* development, which lacks a Smo homolog—could there be a connection with sterol transport? *C. elegans* is a cholesterol auxotroph because it lacks enzymes required for the de novo synthesis of cholesterol and related sterols and relies instead on exogenously supplied sterols to support molting, dauer formation, reproductive growth and locomotion (Chitwood, 1999; Merris et al., 2004). Imaging studies using dehydroergosterol and filipin have suggested that sterols accumulate in the pharynx, nerve ring, excretory gland cell and on the apical surfaces of the gut and spermatozoa (Matyash et al., 2001); however, these techniques have limited sensitivity. Nonetheless, the low abundance of sterols in worms precludes their ability to be major constituents of cell membranes (Kurzchalia and Ward, 2003). In worms, 7-DHC, the vitamin D precursor, is the major endogenous sterol (Chitwood et al., 1983). Given that PTC-3 requires an intact RND transporter domain for function and the evidence that Ptch1 is capable of transporting vitamin D3, it is possible to speculate that PTC-3 is involved in the transport or trafficking of a cholesterol derivative, such as 7-DHC or a lipid, which is needed for hypodermal or cuticle development. Such a model would explain, in part, the molting and membrane defects observed in animals with reduced *ptc-3* activity.

Additional evidence supporting a role for PTC-3 in sterol or lipid transport function comes from our studies showing that expression of *ptc-3* is specifically required in the hypodermis. Rescue of the *ptc*-*3*(*ok121*) mutant phenotype was achieved not only through expression of a wild-type *ptc-3::gfp* translational reporter, but also when *ptc-3::gfp* expression was driven from either the *let-60* or *dpy-7* gene promoter, both of which are active in the hypodermis. Most revealingly, *dpy-7* expression is confined to the hypodermis (Gilleard et al., 1997). With regard to sterol transport, perfusion experiments in *Ascaris suum*, which is also a cholesterol auxotroph, have shown that the hypodermis, and not the intestine, is the primary route of cholesterol absorption (Fleming and Fetterer, 1984). In *C. elegans*, it has been suggested that *lrp-1*, which encodes the worm protein most closely related to vertebrate gp330/megalin, could be a receptor for sterols that are endocytosed by the major hypodermal syncytium hyp7, based on its loss-of-function phenotype and homology to gp330/megalin (Yochem et al., 1999). *lrp-1* and *ptc-3* both express membrane proteins localized to the apical surface of hypodermal cells; in addition, a reduction in the function of either gene impairs the completion of molting, although the likely null phenotype of *ptc-3* is more severe and results in lethality. Given these similarities, it is possible to speculate that LRP-1 promotes exogenous sterol uptake and PTC-3 promotes sterol transport or trafficking.

These studies also indicate that ectopic expression of *ptc-3::gfp*, achieved through the use of heterologous promoters, does not appear to be detrimental to *C. elegans* growth or morphogenesis with the potential exception of vulval development in the case of *let-60p::ptc-3::gfp*. However, as discussed above, the vulval morphogenesis defect could also be a consequence of promoter squelching. In addition, the failure to rescue *ptc-3* mutants through the expression of a *lin-48p::ptc-3::gfp* transgene indicates that the absence of *ptc-3* in the excretory duct cell alone is unlikely to be the primary cause of the osmoregulation defect observed in mutants. Instead, we speculate that the ability of the *dpy-7p::ptc-3::gfp* transgene to rescue a *ptc-3* mutant points to the general importance of the hypodermis in maintaining osmoregulation and in vesicle trafficking during cuticular secretion (Michaux et al., 2000; Liégeois et al., 2007).

### The role of Hh-related and PTC proteins in *C. elegans*

Studies in *C. elegans* have clearly established that PTC proteins have developmental activities that extend beyond their roles as receptors for Hedgehog in other organisms. Morphological defects have been observed when *ptc-3* activity is depleted, suggesting that *ptc-3* could play a role in cell patterning although there is no evidence indicating that *ptc-3* participates in a signal transduction pathway directly regulating cell fate. Is it possible that PTC-3 interacts with the Hh-related proteins? We have shown that RNAi depletion of a subset of *hh-*r genes produced phenotypes resembling those of *ptc-3*(*RNAi*) and *ptr*(*RNAi*) (Zugasti et al., 2005). In addition, analyses of the *hh-*r *wrt-5* and *qua-1* genes have shown that they are expressed in the hypodermis and that their absence results in molting and hypodermal defects (Aspock et al., 1999; Hao et al., 2006a,b,c). Taken together, these studies suggest that the *ptc-3*, *ptr* and *hh-*r genes might participate in similar processes, particularly with regard to hypodermal development. However, at this level of phenotypic analysis, genetic arguments would predict that the *C. elegans* Hh-related proteins function as agonists, not antagonists of PTC and/or PTR activity. How can this apparent genetic problem be reconciled without changing the nature of the potential ligand from antagonist to agonist? First, the existence of this large number of *C. elegans ptr* and *hh*-r genes makes it possible to speculate that these genes participate in a network of interactions. Second, the dynamic pattern of PTC-3::GFP expression raises the possibility that PTC-3 could be required during specific developmental windows, but not constitutively. Thus, it becomes possible to speculate that Hh-r proteins might downregulate subsets of PTR proteins in order to allow other members of this large family to be active. By this scenario, Hh-r proteins would retain an antagonistic role, and the absence of *hh-r* or *ptr* gene could produce similar phenotypes.

Alternatively, if Hh-r and PTC/PTR proteins do not share a ligand/receptor relationship, then it remains possible that the PTC and/or PTR proteins are involved either directly or indirectly in the trafficking of Hh-r peptides. Endocytosis defects have been observed after RNAi has been performed with a subset of *ptr* genes (Zugasti et al., 2005); such defects could potentially lead to the mislocalization of a protein needed for Hh-r secretion. It is likely that insights into the nature of any interaction, or lack thereof, between the Hh-r and PTC and PTR will be gained as more is learned about the mode of Hh-r secretion. Already, it has been shown that a *C. elegans* V0-ATPase subunit, VHA-5, promotes the apical secretion of Hh-r peptides (Liégeois et al., 2006).

Thus, our studies have shown that *ptc-3* is an essential *C. elegans* gene that has evolved to have a developmental role in *C. elegans* that is distinct from *ptc-1*. Although neither *C. elegans ptc-1* nor *ptc-3* plays any role in regulating a Smo homolog, the finding that the permease transporter GxxxD motif is essential for the activity of both the *C. elegans* PTC-3 and human Patched proteins suggests that the movement of lipids or sterols could represent a fundamental and evolutionarily conserved function for these proteins.

The following are the supplementary materials related to this article.

Supp. Fig. 1 Alignment of SCAP and Ptc proteins across phyla showing the position of an invariant Asp (D) residue in the SSD. Overhead line shows extent of TM region. Alignment is visualized using Jalview (Clamp et al., 2004).

Supp. Fig. 2 Expression of PTC-3(SSD, D780N)::GFP in *ptc-3; crEx234* homozygotes. (A, B) Expression in the hypodermis of embryos prior to hatching. (C, D) Expression in the excretory duct. (E, F) L3 larval stage expression in the uterine utse cell, lateral view. Images on left are DIC Nomarski micrographs, images on the right are the same as those on the left illuminated with epi-fluorescence to visualize GFP. Scale bars, 10 μM.

Supp. Fig. 3 Clustal W alignment of the Human PTCH1 and the *C. elegans* PTC and PTR proteins, highlighting the GxxxD motif associated with the permease transporter domains found in TM4 and TM10. Overhead line shows extent of the TM region. Alignment is visualized using Jalview (Clamp et al., 2004).

Supp. Fig. 4 Expression of PTC-3(GxxxD, D697A)::GFP in *crEx236* animals. (A, B) Expression in the hypodermis of embryos prior to hatching. (C, D) Expression in the excretory duct. (E, F) L3 larval stage expression in the uterine utse cell, lateral view. Images on left are DIC Nomarski micrographs, images on the right are the same as those on the left illuminated with epi-fluorescence to visualize GFP. Scale bars, 10 μM.

## Figures and Tables

**Table 1:** Transgenic arrays capable of rescuing the embryonic lethality of *ptc-3* homozygotes.

P_0_ genotype	*N*^a^	Adultsb,c	Dead eggs^b^
*ptc-3*(ds*RNAi*) d	5	15.4 ± 4.5	133 ± 48.0
*ptc-3/+*	15	210 ± 37.5	59.7 ± 11.2
*ptc-3; crEx40* [*ptc-3p::ptc-3::gfp*]	5	133.2 ± 10.5 (49%)	136.2 ± 27.5 (51%)
*ptc-3; crEx442* [*let-60p::ptc-3::gfp*]	9	94 ± 22 (50%)	92.8 ± 24.3 (50%)^e^
*ptc-3; crEx437* [*dpy-7p::ptc-3::gfp*]	5	85 ± 12 (41%)	123.2 ± 21.5 (49%)^e^
*ptc-3;crEx234* [*ptc-3p::ptc-3(D789N)::gfp*]	6	46.5 ± 9.9 (19%)	199.7 ± 9.9 (81%)

a *N* refers to the number of P_0_ animals scored.

b Numbers represent mean ± standard deviation (SD); corresponding percentages are found in brackets.

c The number of homozygous viable *ptc-3* adults rescued by a given extrachromosomal array is consistent with its transmission frequency (40–50% for *crEx40, crEx442* and *crEx437*; 15–20% for *crEx234*).

d By contrast, *rhr-1*(ds*RNAi)* control animals (*N* = 6) displayed no obvious phenotype and produced 145 ± 11 adults and 1.0 ± 0.9 dead eggs.

e Numbers include both dead eggs and dead larvae; see text for additional details.
