# Supplementary material for: C. elegans patched-3 is an essential gene implicated in osmoregulation and requiring an intact permease transporter domain
Source: Dev Biol. 2011 Mar 15;351(2-4):242–53. doi: 10.1016/j.ydbio.2010.12.035 (PMC3078328; doi:10.1016/j.ydbio.2010.12.035)
Supplement: Supp. Fig. 1 — Alignment of SCAP and Ptc proteins across phyla showing the position of an invariant Asp (D) residue in the SSD. Overhead line shows extent of TM region. Alignment is visualized using Jalview (Clamp et al., 2004). [file mmc1.pdf]

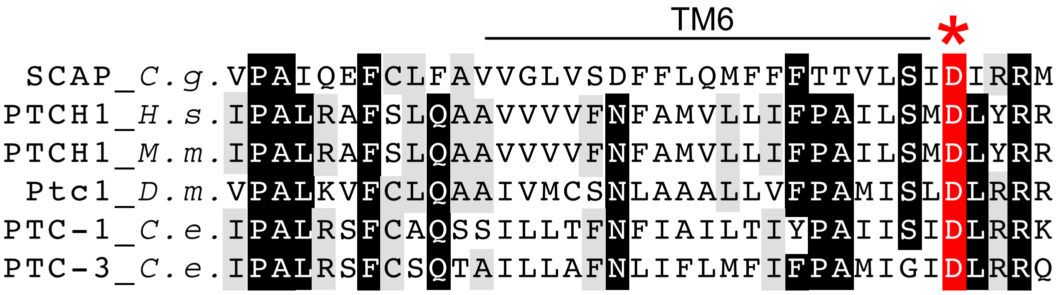
TM6
*
SCAP_C.g. VPAIQEFCLFAVVGLVSDFFLQMFFFTTVLSIDIRRM
PTCH1_H.s. IPALRAFSLQAAVVVVFNFAMVLLIFPAILSMDLYRR
PTCH1_M.m. IPALRAFSLQAAVVVVFNFAMVLLIFPAILSMDLYRR
Ptc1_D.m. VPALKVFCLQAAIVMCSNLAAALLVFPAMISLDLRRR
PTC-1_C.e. IPALRSFCAQSSILLTFNFIAILTIYPAIISIDLRRK
PTC-3_C.e. IPALRSFCSQTAILLAFNLIFLMFIFPAMIGIDLRRQ

Suppl. Fig. 1

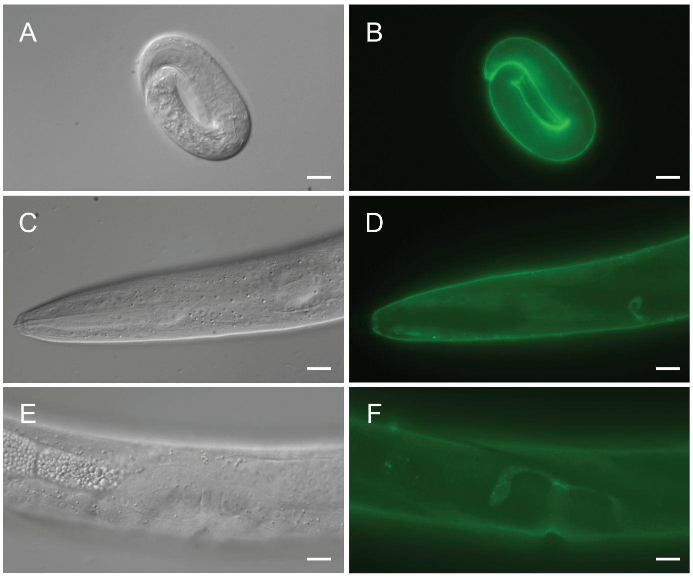

Suppl. Fig. 2

```
                             GxxxD/E
                                                  TM4
Hs-PTCH1          NAATTQVLPFLALGVGVDDVFLLA
Ce-PTC-1          NAATTQIVPFLTLGIGVDNMFMLL
Ce-PTC-3          NAATTQVVPFLSLGLGIDDMFLLL
Ce-PTR-1          NAIVN-VSPFLILCIGIDDLFIMC
Ce-PTR-2          NDIVG-VMPFLVLAVGVDNMFLMV
Ce-PTR-3          GSILC-VTPFLVLAIGVDDSYLMA
Ce-PTR-4          NPVTS-TMPFLVLAVGVDDDFLMM
Ce-PTR-5          NSFML-VMPFLVMGIGVDSCFLMI
Ce-PTR-6          AEITL-IAPFLVLSIGVDDMFIAV
Ce-PTR-7/DAF-6    NVINT-IIPFLIIAIGIDDMFLMN
Ce-PTR-8          SPIMC-ITPFLVLAISVDDSFLML
Ce-PTR-9          LPLVF-IMPFLVVSIGTDNMFLML
Ce-PTR-10         NDIIA-VMPFLVVAVGTDNMFLMV
Ce-PTR-11         FSIQC-VTPFLVLGIGVDDAFILL
Ce-PTR-12         GSILC-VTPFLVLAIGVDDAYLMI
Ce-PTR-13         NSIMC-ITPFLILGIGVDDAFLLL
Ce-PTR-14         GTILC-VTPFLILAIGVDDAYLQV
Ce-PTR-15         INQVT-VMPFIALAIGVDDVYVML
Ce-PTR-16         NSITL-VMPFLIIGVGVDDVFIII
Ce-PTR-17         VDMCT-VMPFLSLTIGIDDTFLML
Ce-PTR-18         LPIVT-VVPFLILSIGVDDVFIFI
Ce-PTR-19         TSVAY-SMPFIVFSVGVDNVFILL
Ce-PTR-20         SNILT-VVPFLVVTIGIDDAFLIL
Ce-PTR-21         NSPMM-IMPFLINGIGVNDAFLTL
Ce-PTR-22         QSIIV-STLFLVIAIGIDDIFIIL
Ce-PTR-23         ASILC-VIPFLVLSIGVDSSYLMI
Ce-PTR-24         TPLII-FTPFLALIHGYYTVIMLT
Ce-CHE-14         PLLNL-VTFVLLIAIGSDDAFLLK

                         GxxxD/E          TM10
Hs-PTCH1          LSAVPVVILIASVGIGVEFTVHVA
Ce-PTC-1          LNPVSAVTLITAVGIGVEFTVHVV
Ce-PTC-3           NPISAVTLICAVGIGVEFTAHVE
Ce-PTR-1          LDSVSIITVIMCIGFSVDLSAHIA
Ce-PTR-2          LDAISMITIIMSIGFSVDYSAHIA
Ce-PTR-3          LDPIMMSATVMSIGFSVDIPSHVS
Ce-PTR-4          LDSVSMGCIVMAIGLAVDYSVHIC
Ce-PTR-5          LDPVVMAAVLMSIGLSVDFIAHVA
Ce-PTR-6          LDPISMITIIMSIGFS EFSAHIT
Ce-PTR-7/DAF-6    LDAVSMISIIMSIGFAVDLSAHII
Ce-PTR-8          LDPVIMSIAIMCIGFSVDIPAHVA
Ce-PTR-9          LDIISMITIVMSVGFSVDYVTHTT
Ce-PTR-10         LDAISMITIIMSVGFSVDYSAHIT
Ce-PTR-11         LDPVIQVDVLLATGFSVDYTAHVA
Ce-PTR-12         LDPIVMSALIISIGFSVDIPAHIS
Ce-PTR-13         LDPVTMIDVIMAIGFSVDYSAHVC
Ce-PTR-14         LDPISMACLIMSIGFSVDFPAHIT
Ce-PTR-15         LDPMTVVNILMSIGQC DFATHVG
Ce-PTR-16         LDPITMCTTLMSIGFSVDFTAHIS
Ce-PTR-17         LDATSMITVAMSVGFSVDFAAHVS
Ce-PTR-18         LDPISMTTLLMAIGFSVDFVAHIT
Ce-PTR-19         LDFISMVTIVMSIGFCVDFAAHLA
Ce-PTR-20         LDAVSLISMLMSIGFSVDYSAHVC
Ce-PTR-21         LDPVTLCAVIVSIGMSVDFVAHVA
Ce-PTR-22         M PVSTASLLMSIGFSVDISAHIS
Ce-PTR-23         LDPITMAAMIISIGFSVDIPAHVS
Ce-PTR-24         INPFNAAFFLIIAALA SCTTHYC
Ce-CHE-14         LGVLEAVILVLVVGLSFDYTLHYG
```

Suppl. Fig. 3

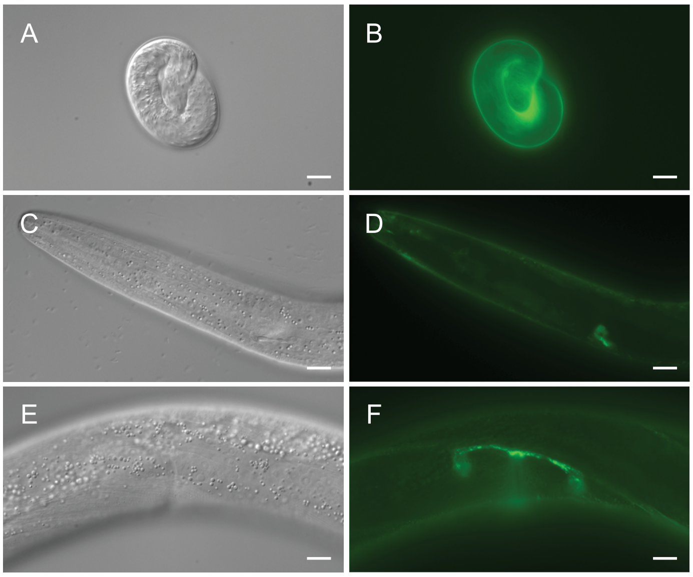

Suppl. Fig. 4
